# The Use of a Combined Bioinformatics Approach to Locate Antibiotic Resistance Genes on Plasmids From Whole Genome Sequences of *Salmonella enterica* Serovars From Humans in Ghana

**DOI:** 10.3389/fmicb.2018.01010

**Published:** 2018-05-17

**Authors:** Egle Kudirkiene, Linda A. Andoh, Shahana Ahmed, Ana Herrero-Fresno, Anders Dalsgaard, Kwasi Obiri-Danso, John E. Olsen

**Affiliations:** ^1^Department of Veterinary and Animal Sciences, Faculty of Health and Medical Sciences, University of Copenhagen, Copenhagen, Denmark; ^2^Department of Theoretical and Applied Biology, Kwame Nkrumah University of Science and Technology, Kumasi, Ghana

**Keywords:** whole genome sequencing, *Salmonella*, multidrug resistance, plasmids, Ghana

## Abstract

In the current study, we identified plasmids carrying antimicrobial resistance genes in draft whole genome sequences of 16 selected *Salmonella enterica* isolates representing six different serovars from humans in Ghana. The plasmids and the location of resistance genes in the genomes were predicted using a combination of PlasmidFinder, ResFinder, plasmidSPAdes and BLAST genomic analysis tools. Subsequently, S1-PFGE was employed for analysis of plasmid profiles. Whole genome sequencing confirmed the presence of antimicrobial resistance genes in *Salmonella* isolates showing multidrug resistance phenotypically. ESBL, either *bla*_TEM52−B_ or *bla*_CTX−M15_ were present in two cephalosporin resistant isolates of *S*. Virchow and *S*. Poona, respectively. The systematic genome analysis revealed the presence of different plasmids in different serovars, with or without insertion of antimicrobial resistance genes. In *S*. Enteritidis, resistance genes were carried predominantly on plasmids of IncN type, in *S*. Typhimurium on plasmids of IncFII(S)/IncFIB(S)/IncQ1 type. In *S*. Virchow and in *S*. Poona, resistance genes were detected on plasmids of IncX1 and TrfA/IncHI2/IncHI2A type, respectively. The latter two plasmids were described for the first time in these serovars. The combination of genomic analytical tools allowed nearly full mapping of the resistance plasmids in all *Salmonella* strains analyzed. The results suggest that the improved analytical approach used in the current study may be used to identify plasmids that are specifically associated with resistance phenotypes in whole genome sequences. Such knowledge would allow the development of rapid multidrug resistance tracking tools in *Salmonella* populations using WGS.

## Introduction

The increased and widespread use of antimicrobials in human and veterinary medicine has resulted in increased levels of antimicrobial resistance (World Health Organization, 2014). Global trade and travel mean that antimicrobial resistant bacteria that emerge in one country easily spread to other countries (Aarestrup et al., 2007; Hendriksen et al., 2011; Le Hello et al., 2011), and in recent years, multidrug and cephalosporin-resistant *Salmonella* have become a public health concern worldwide (Su et al., 2004; Yan et al., 2005; Anjum et al., 2011). This is problematic, since third generation or extended-spectrum cephalosporins are the drugs of choice in the treatment of *Salmonella* infections when fluoroquinolone treatment is unsuccessful (Parry and Threlfall, 2008).

The most common mean of antibiotic resistance development in *Salmonella* is through the acquisition of mobile genetic elements such as plasmids, transposons and integrons carrying resistance genes. The major concern is that these mobile elements, in particular plasmids, spread rapidly in the population, thus posing a risk of emergence of highly resistant clones of *Salmonella* within both human and animal hosts (Hoffmann et al., 2017).

Complete plasmid sequencing from *Salmonella* isolates from various sources is the ideal method to understand the nature and transfer of antibiotic resistance in bacteria. Though plasmid sequencing remains the main strategy for complete plasmid characterization, plasmid extraction from whole genome sequences is becoming increasingly common, and to date, a number of bioinformatic tools have been developed for *in silico* detection and characterization of plasmids in whole genome sequences (Arredondo-Alonso et al., 2017; Orlek et al., 2017). However, none of these tools (e.g., PlasmidFinder, PlasmidSPAdes, Recycler, PLACNET, etc.) are sufficiently powerful if used alone, and there is a need to develop combined approaches to allow confident identification and characterization of plasmids *in silico*.

In Ghana, *Salmonella* and other Enterobacterioceae which are resistant to cephalosporins have been reported in humans (Mills-Robertson et al., 2003; Obeng-Nkrumah et al., 2013; Andoh et al., 2017), but little is known about the plasmids that carry genes coding for such resistances. In this study, a strategic combination of bioinformatics tools were applied on whole genome sequence data to identify plasmids harboring genes associated with extended-spectrum β-lactamases (ESBLs) and other antimicrobial resistance phenotypes in multidrug-resistant and putatively ESBL-producing *Salmonella enterica* isolates from humans in Ghana.

## Methods

### Strain collection

Sixteen antibiotic resistant *Salmonella* isolates were obtained from a previous study on human salmonellosis in Ghana. Strains were previously assigned to a specific serovar and antimicrobial resistance pattern (Andoh et al., 2017). Strains and their relevant characteristics are listed in Table 1.

**Table 1:** *Salmonella enterica* isolates included in the study and their relevant characteristics.

**Strain ID**	**Serovar**	**ST type**	**AR phenotype^a^**	**AR genes identified (ResFinder)**
113	*S*. Enteritidis	ST11	**AMP/AMX**	***bla**_***TEM*****−1*****B***_*
15	*S*. Enteritidis	ST11	**AMP/AMX/TET/TMP/SMZ**	***bla**_***TEM*−1*B***_**/tet(A)/dfrA15/sul1**/strA/strB*
102	*S*. Enteritidis	ST11	**AMP/AMX/TET/TMP/SMZ**	***bla**_***TEM*−1*B***_**/tet(A)/dfrA15/sul1**/cat/strA/strB*
14	*S*. Enteritidis	ST11	**AMP/AMX/TET/TMP/**SMZ	***bla**_***TEM*−1*B***_**/tet(A)/dfrA15/**cat/strA/strB*
73	*S*. Enteritidis	ST11	**AMP/AMX/TET/TMP/SMZ**	***bla**_***TEM*−1*B***_**/tet(A)/dfrA15/sul1/**catA1/strA/strB*
62	*S*. Enteritidis	ST11	**AMP/AMX/TET/TMP/SMZ**	***bla**_***TEM*−1*B***_**/tet(A)/dfrA15/sul1**/strA/strB*
4233	*S*. Typhimurium	ST313	**AMP/AMX/TMP/SMZ/CHL**	***bla**_***TEM*−1*B***_**/dfrA15/sul1/sul2/catA1**/strA/strB/addA1*
2256	*S*. Typhimurium	ST313	**AMP/AMX/TET/TMP/SMZ/CHL**	***bla**_***TEM*−1*B***_**/tet(A)/dfrA15/sul1/sul2/catA1**/strA/strB/addA1*
4829	*S*. Typhimurium	ST313	**AMP/AMX/TMP/SMZ/CHL**	***bla**_***TEM*−1*B***_**/dfrA15/sul1/sul2/catA1**/strA/strB/addA1*
B48	*S*. Typhimurium	ST313	**AMP/AMX/TMP/SMZ/CHL**	***bla**_***TEM*−1*B***_**/dfrA15/sul1/sul2/catA1**/strA/strB/addA1*
44	*S*. Typhimurium	ST19	AMP/SMZ	NI^b^
590	*S*. Virchow	ST16	**AMP/AMX/CT-CTL/**NAL	***bla**_***TEM*−52*B***_*
645	*S*. Virchow	ST359	AMP	*cat*
4770	*S*. Colindale	ST584	AMP/SMZ	NI
114	*S*. Oakland	ST605	AMP	NI
323	*S*. Poona	ST308	**AMP/AMX/CT-CTL/TET/ CIP/TMP/SMZ/GEN**	***bla**_***TEM*−1*B***_**/bla**_***CTX*−*M*−15**_**/bla**_***OXA*−1**_**/QnrB1/aac(6')Ib-cr/ tet(A)/dfrA15/sul2**/catB3/strA/strB/**aac(3)-Iia***

*The serovars and the antimicrobial resistance phenotypes were previously described (Andoh et al., 2017). Serovars were confirmed in silico in the current study*.

a *Amp, ampicillin; Amx, amoxicillin; Tet, tetracycline; Tmp, trimethoprim; Smz, sulphometoxazole; Chl, chloramphenicol; Ct/Ctl, cefotaxime - cefotaxime + clavulanic acid; Nal, nalidixic acid; Cip, ciprofloxacin; Gen, gentamicin; bold, agreement between the phenotype and genotype*.

b *No antimicrobial resistance genes were identified*.

### Phenotypic test for ESBL

ESBL phenotype was determined using ESBL CT/CTL 16/1 ETEST® for Antimicrobial Resistance Detection (BioMerieux, France). Manufacturer's instructions were followed to perform the test and to interpret the results.

### Whole genome sequencing

The 16 *Salmonella* isolates were grown in Luria broth (LB) (240230; Difco, USA) for 16 h at 37°C while shaking, and genomic DNA was isolated using a blood and tissue kit (Qiagen, Stockach, Germany) according to the instructions of the supplier. Genomes were sequenced at a 300-bp paired-end-read format using the Nextera XT library preparation kit and the MiSeq instrument (Illumina, San Diego, California, USA) as previously reported (Madoshi et al., 2016). CLC Genomic Workbench v6.5 software package (https://www.qiagenbioinformatics.com/products/clc-genomics-workbench/) was used for sequence-reads quality-assessment and adapter trimming. Draft genomes assembling was performed with SPAdes v3.10.1 (Bankevich et al., 2012), and the quality of the assembly was evaluated with QUAST v.2.3 (Gurevich et al., 2013). The draft genome sequences from the strains were submitted to GenBank (BioProject ID: PRJN335586).

### *In silico* analysis of serovar, serogroup and antigenic profile

SISTR (Yoshida et al., 2016) and SeqSer (Zhang et al., 2015) bioinformatics tools were used to identify the sequence type (ST type), serovar, serogroup and antigenic profile in the 16 draft genomes of *Salmonella*.

### *In silico* plasmid identification

The presence of plasmids in the genomes was identified using a combination of the following bioinformatic tools: PlasmidFinder (Carattoli et al., 2014), plasmidSPAdes v3.10.1 (Antipov et al., 2016), BlastN at NCBI and locally within the CLC Genomics Workbench v6.5. Briefly, in the first step of the analysis, PlasmidFinder was used to identify plasmid replicon location in the whole genome assembly generated by SPAdes. To improve plasmid detection and to identify all contigs representing a specific plasmid, the plasmidSPAdes tool was then employed. The algorithm in plasmidSPAdes is able to predict which contigs belong to plasmid DNA, and to assign those contigs into components. Each component is considered as a putative plasmid composed of one or more contigs. In the third step, components (putative plasmids, SPAdes output) containing specific plasmid replicons and their combinations (PlasmidFinder output) from one selected strain were used to search for the most similar/reference plasmids at NCBI nr-database using BlastN. Finally, the sequences of the identified reference plasmids were downloaded from GenBank and searched back in all strains using local BlastN in CLC Genomics Workbench and by progressive MAUVE (Darling et al., 2010). This allowed the identification of plasmid DNA (contigs), which could not be assigned to a specific plasmid replicon type and/or its component by PlasmidFinder and plasmidSPAdes. A graphic overview of the approach giving an example of the analysis of one selected isolated is shown in Supplementary Figure 1.

### *In silico* identification of antibiotic resistance genes and their association with plasmids

ResFinder (Zankari et al., 2012), available on the Centre for Biological Sequence analysis (CBS) server^1^ was employed for the *in silico* prediction of acquired antibiotic resistance genes in the genomes under study. For a hit to be reported by ResFinder, it had to cover at least 60% of the length of the gene sequence in the database with a sequence identity of 60%. The prediction of location of antibiotic resistance genes in plasmids was performed in a step-wise procedure by combining the results of resistance prediction and plasmid identification steps (Supplementary Figure 1). The output from ResFinder gives the contig IDs on which antimicrobial resistance genes are located in the whole genome assembly generated by SPAdes. Contigs identified by ResFinder were searched to determine whether they were the same as those carrying plasmid replicons identified using PlasmidFinder or those assigned to specific components created by plasmidSPAdes. Contigs, which carried antimicrobial resistance genes, but which were not assigned to plasmids by using the above tools, were blasted against reference plasmids locally.

### S1-PFGE plasmid profiling

Plasmid profiling was performed according to the protocol described by Barton et al. (1995). Briefly, plasmids were digested with the S1 nuclease, and their linear forms were visualized using Pulsed-field gel electrophoresis (PFGE). The plasmid sizes were estimated by comparing their electrophoretic mobility with the electrophoretic mobility of fragments generated by *XbaI* digestion of *Salmonella* Braenderup strain H9812^2^.

## Results

### *In silico* confirmation of serovar and predicition of sequence type

The 16 strains under study belonged to six serovars [*S*. Enteritidis (*n* = 6), *S*. Typhimurium (*n* = 5), *S*. Virchow (*n* = 2), *S*. Colindale (*n* = 1), *S*. Poona (*n* = 1), *S*. Oakland (*n* = 1)], of *Salmonella enterica* according to the phenotypic characterization previously performed (Andoh et al., 2017). All serovars were confirmed genotypically using the combination of SeqSero and draft genome assemblies analysis tools available on the SISTR platform (Table 1 and Supplementary Table 1).

Based on sequence type prediction by SISTR, all *S*. Enteritidis isolates were assigned to the same sequence type (ST11). The majority of *S*. Typhimurium isolates (*n* = 4/5) belonged to ST313, and only the *S*. Typhimurium strain 44 was assigned to ST19. The two *S*. Virchow, one *S*. Colindale, one *S*. Poona, and one *S*. Oakland isolates were predicted to belong to distinct ST types (Table 1).

### *In silico* prediction of antimicrobial resistance genes

The majority (81.3%) of the 16 strains selected for the whole genome sequence analysis were phenotypically resistant to more than one antimicrobial (Andoh et al., 2017). With regards to resistance to β-lactams, two out of the 16 strains showed resistance to cefotaxime/cefotaxime + clavulanic acid, and thus were ESBL-positive. The remaining 14 strains were only resistant to amino-penicillins (Table 1). The susceptibility of these strains to antimicrobials, as determined phenotypically (Andoh et al., 2017) was related to the presence of resistance genes in the whole genome sequences using the ResFinder tool.

Among the 16 *Salmonella* strains analyzed, ResFinder detected the presence of *bla*_TEM−1B_ in 11 amino-penicillin resistant isolates. Two isolates associated with an ESBL phenotype carried recognized ESBL genes *bla*_TEM−52B_ and *bla*_CTX−M−15_, respectively (Table 1). Five or more other antimicrobial resistance genes were identified per isolate from the genome sequences, with the most common genes encoding resistance to trimethoprim (*dfrA1/dfrA15)*, aminoglycosides *(strA/strB/aadA1)*, sulfamethoxazole *(sul1/sul2)*, chloramphenicol *(cat/catA1/catB3)* and tetracycline (*tetA*). The majority of the phenotypic resistances were confirmed by the genome sequence analysis [Sul (9/12; 75.0%), Tmp (10/10; 100.0%), Tet (7/7; 100.0%), Amp-Amx (12/16; 75.0%), Chl (4/4; 100%), Cip (1/1; 100.0%), Nal (0/1; 0.0%), Gen (1/1; 100.0%)] (Table 1). The streptomycin-resistance genes *strA/strB* and the streptomycin/spectinomycin resistance gene *aadA1* were detected in 10 and 4 out of the 16 strains, respectively. These antimicrobials were not screened phenotypically.

### *In silico* prediction of plasmids and plasmid replicons

PlasmidFinder was first used to identify plasmid replicons in the 16 *Salmonella enterica* strains under study. All genomes of *S*. Enteritidis (*n* = 6) and *S*. Typhimurium (*n* = 5) carried plasmid replicons of the IncFII(S) and IncFIB(S) types (Table 2). In addition, IncN and IncX1 type plasmid replicons were found in five and three isolates of *S*. Enteritidis, respectively, and all isolates of *S*. Typhimurium, except one, carried plasmid replicons of the ColRNAI type. Four isolates of *S*. Typhimurium harbored a plasmid replicon of the IncQ1 type. One isolate of *S*. Virchow showed a plasmid replicon of the IncX1 type. The *S*. Poona strain and another *S*. Virchow isolate carried three plasmid replicons of the IncHI2, IncHI2A and TrfA type (Table 2). PlasmidFinder did not detect any plasmid replicons in the isolates of *S*. Colindale and *S*. Oakland.

**Table 2:** Performance of bioinformatic tools in respect to plasmid reconstruction and detection of antimicriobial resistance gene location in the whole genome sequences of the *Salmonella* strains under study.

**Serovar strain ID**	**Plasmid type**	**Plasmid replicon/component detection**		**Other tools needed for AR-association and full plasmid reconstruction**	**Plasmid profiling using S1-PFGE**
		**Plasmid finder**	**Plasmid SPAdes**		
***S***. **Enteritidis**					
14	IncFII/IncFIB	+/+	–/–	NI^a^	+
62	IncFII/IncFIB	+/+	+/+	NI	+
102	IncFII/IncFIB	+/+	–/–	NI	+
73	IncFII/IncFIB	+/+	–/+	NI	–^b^
15	IncFII/IncFIB	+/+	–/–	NI	+
113	IncFII/IncFIB	+/+	–/–	BlastN	+
***S***. **Enteritidis**					
14	IncN	+	+	ResFinder	+
62	IncN	+	+	ResFinder	+
102	IncN	+	+	ResFinder	+
73	IncN	+	–	BlastN	–^b^
15	IncN	–	+	BlastN	+
113	IncN	–	–	NI	–
***S***. **Enteritidis**					
62	IncX1	+	+	NI	–
102	IncX1	+	+	NI	–
73	IncX1	+	+	NI	–
***S***. **Typhimurium**					
4233	IncFII/IncFIB/IncQ1	+/+/+	+/+/+	ResFinder/BlastN	+
4829	IncFII/IncFIB/IncQ1	+/+/+	+/+/+	NI	+
2256	IncFII/IncFIB/IncQ1	+/+/+	+/+/+	ResFinder	+
B48	IncFII/IncFIB/IncQ1	+/+/+	–/–/–	ResFinder/BlastN	+
44	IncFII/IncFIB/IncQ1	+/+/–	–/+/–	NI	+^c^
***S***. **Typhimurium**					
4233	ColRNAI	+	+	NI	–
4829	ColRNAI	+	+	NI	–
2256	ColRNAI	+	+	NI	–
B48	ColRNAI	+	+	NI	–
***S*****. Virchow**					
590	IncX1	+	+	ResFinder	+
***S*****. Virchow**					
590	TrfA/IncHI2/IncHI2A	+/+/+	+/+/+	NI	+
***S***. **Poona**					
323	TrfA/IncHI2/IncHI2A	+/+/+	–/–/+	BlastN	+

a *no antimicrobial resistance genes identified*.

b *a fragment of 100 kb corresponding to the size of both IncFII(S)/IncFIB(S) and IncN detected*.

c *IncQ absent*.

PlasmidFinder does not report plasmid replicons, unless they are present in the PlasmidFinder database. In an attempt to overcome this limitation, plasmids were also searched with the plasmidSPAdes tool, which uses the read coverage of contigs to assist in distinguishing between plasmids and the chromosome. In *S*. Enteritidis, the contigs predicted by PlasmidFinder to carry either IncFII(S) or IncFIB(S) plasmid replicons were confirmed in only two strains with SPAdes (Table 2). In one strain, both replicons were connected into one component, which indicated that they originated from the same plasmid (data not shown). Plasmid contigs with replicons of IncN type were confirmed in four strains, but failed in one strain. In two strains, IncN and IncX1 replicons were found on the same SPAdes component, whereas in other two strains IncN was found alone (data not shown). In *S*. Typhimurium, plasmid replicons IncFII(S) and IncFIB(S) were confirmed by SPAdes in four strains (Table 2). In one strain, sequences containing these replicons were connected into the same component, in two strains they were connected together with the contigs carrying IncQ1 plasmid replicon, and in one strain only the IncFIB(S) replicon was confirmed. PlasmidSPAdes confirmed the presence of ColRNAI containing contigs in all strains. In *S*. Virchow isolate 590, one of the putative plasmids predicted by SPAdes contained two plasmid replicons (TrfA and IncHI2), one putative plasmid contig harbored IncX1, and one putative plasmid harbored IncHI2A (Table 2). Three putative plasmids were predicted using SPAdes in the *S*. Oakland strain, yet no plasmid replicons were previously identified using PlasmidFinder tool. In *S*. Poona, only a contig carrying the IncHI2A replicon type was confirmed. No putative replicon plasmids were found in *S*. Virchow isolate 648, and in the *S*. Colindale strain.

**Figure 1:**
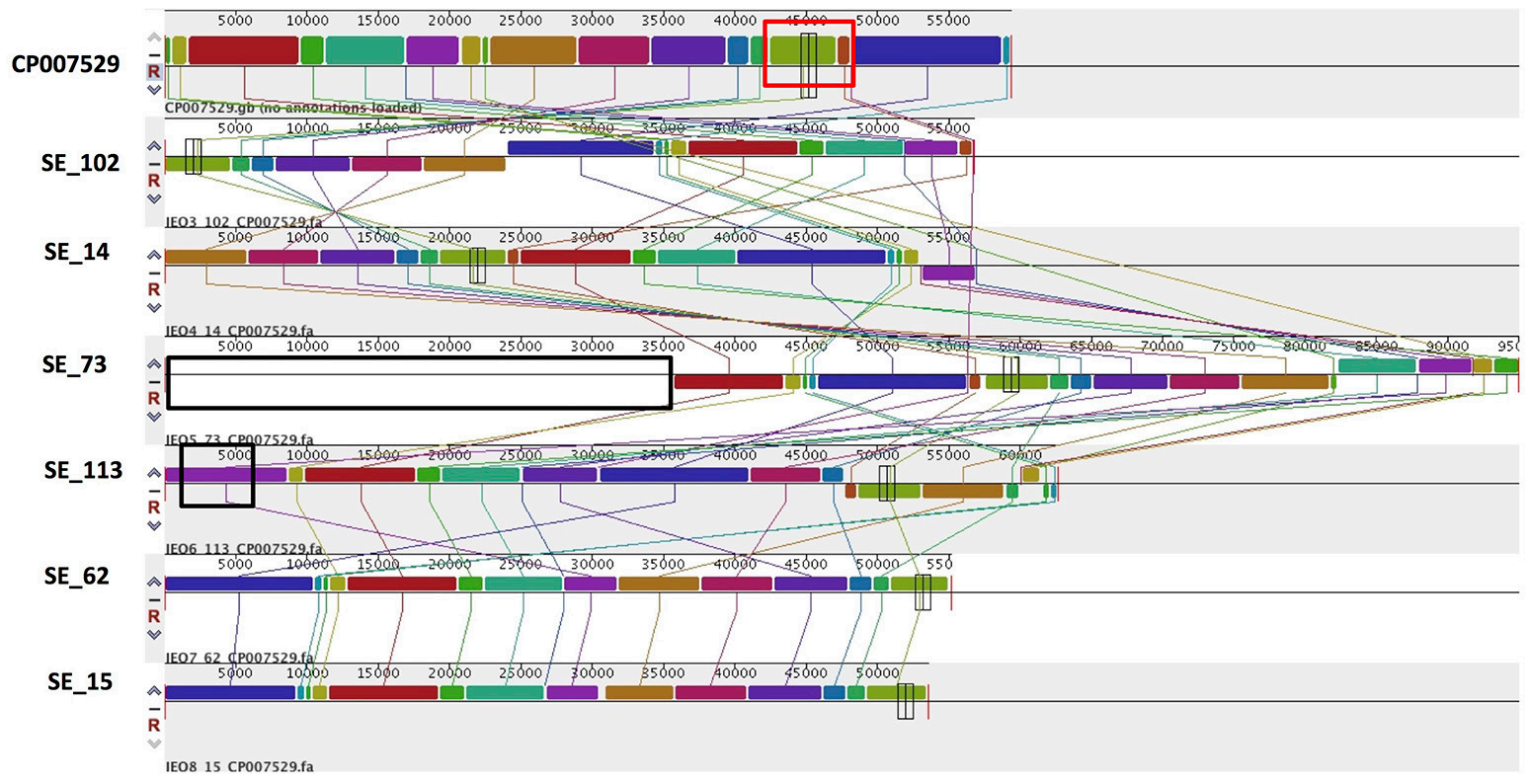
Alignment of IncFII(S)/IncFIB(S) plasmid homologs of *Salmonella* Enteritidis to a reference plasmid CP007529. The alignment was created with MAUVE. Top row shows the reference plasmid, and the rows below the reconstructed plasmids in *Salmonella* Enteritidis from the study. Each of the colored blocks outlines a region of the genome sequence that aligned to part of another genome, and is presumably homologous. Areas with no blocks were not aligned and contain sequence elements specific to a particular genome. The red box indicates the *spv* virulence- associated region in the reference plasmid. Black boxes surrounding a block in SE_113 and in SE_73 indicate antibiotic resistance gene *blaTEM*-1 and predicted IncN plasmid incorporation, respectively.

PlasmidSPAdes components containing specific plasmid replicons were used to search for the most similar reference plasmids at NCBI nr-database using BlastN. The local BlastN and the alignment of the identified reference plasmids-sequences against the draft genome sequences allowed full reconstruction of plasmids in all isolates (Tables 2, 3). This analysis resulted in the detection of three plasmids in the strains of *S*. Enteritidis. The first one carrying plasmid replicons of IncFII(S) and IncFIB(S) was homologous (length coverage of 100%, sequence identity of 92–94%) to a plasmid of *S*. Enteritidis of 59,369 bp in length (GenBank: CP007529). This plasmid corresponds to the *spv*-encoding virulence plasmid of *S*. Enteritidis (Figure 1) (Allard et al., 2013). The second one carrying an IncN plasmid replicon was most similar (length coverage of 100%, sequence identity of 94–96%) to a plasmid of an uncultured bacterium from a wastewater treatment plant in Germany (GenBank: JN102343) of 47,905 bp in length. The third plasmid carried a replicon of the IncX1 type and was similar (length coverage of 100%, sequence identity of 100%) to an *E. coli* plasmid of 1,552 bp (GenBank: CP011143).

**Table 3:** Genome assembly characteristics of *Salmonella enterica* serovars, resistance plasmids and resistance genes identified.

**Strain ID**	**Genome size, Mb**	**No. of contigs^a^**	**Genome coverage^a^**	**Plasmid coverage^b^**	**Plasmids**	**Size *in silico*, bp**	**AR genes on plasmids**
***S***. **Enteritidis**							
113	4.7	187	45	35	IncFII/IncFIB	62,702	*bla_*TEM*−1*B*_*
15	4.7	260	23	25	IncFII/IncFIB	53,542	NI^c^
				40	IncN	48,116	*bla_*TEM*−1*B*_/tet(A)/dfrA15/sul1/strA/strB*
102	4.8	154	53	53	IncFII/IncFIB	56,756	NI
				174	IncN	53,899	*bla_*TEM*−1*B*_/tet(A)/dfrA15/sul1/cat/strA/strB*
14	4.8	189	41	41	IncFII/IncFIB	56,974	NI
				111	IncN	53,817	*bla_*TEM*−1*B*_/tet(A)/dfrA15/cat/strA/strB*
73	4.8	108	60	24	IncFII/IncFIB/IncN	95,014	*bla_*TEM*−1*B*_/tet(A)/dfrA15/sul1/catA1/strA/strB*
62	4.7	179	49	42	IncFII/IncFIB	55,180	NI
				90	IncN	54,571	*bla_*TEM*−1*B*_/tet(A)/dfrA15/sul1/strA/strB*
***S***. **Typhimurium**							
4233	5.0	114	60	25	IncFII/IncFIB/IncQ1	102,170	*bla_*TEM*−1*B*_/dfrA15/sul1/sul2/catA1/strA/strB/addA1*
2256	5.0	172	82	19	IncFII/IncFIB/IncQ1	111,047	*bla_*TEM*−1*B*_/tet(A)/dfrA15/sul1/sul2/catA1/strA/strB/addA1*
4829	4.9	185	54	26	IncFII/IncFIB/IncQ1	108,110	*bla_*TEM*−1*B*_/dfrA15/sul1/sul2/catA1/strA/strB/addA1*
B48	4.8	302	33	20	IncFII/IncFIB/IncQ1	110,306	*bla_*TEM*−1*B*_/dfrA15/sul1/sul2/catA1/strA/strB/addA1*
44	4.8	204	32	22	IncFII/IncFIB	90,695	NI
***S*****. Virchow**							
590	4.8	170	30	44	IncX1	38,603	*bla_*TEM*−52*B*_*
				18	TrfA/IncHI2/IncHI2A	382,887	NI
645	4.6	266	28	NI	NI	NI	NI
***S*****. Colindale**							
4770	4.7	170	43	NI	NI	NI	NI
***S*****. Oakland**							
114	4.6	207	24	NI	NI	NI	NI
***S*****. Poona**							
323	4.7	205	32	25	TrfA/IncHI2/IncHI2A	338,354	*bla_*TEM*−1*B*_/bla_*CTX*−*M*−15_/bla_*OXA*−1_/QnrB1/aac(6')Ib-cr/ tet(A)/dfrA15/sul2/catB3/strA/strB/aac(3)-Iia*

a *number of contigs and their average coverage after the assembly with SPAdes (includes plasmids and chromosome)*.

b *average coverage of contigs belonging to a specific plasmid*.

c *no antimicrobial resistance genes were identified*.

**Figure 2:**
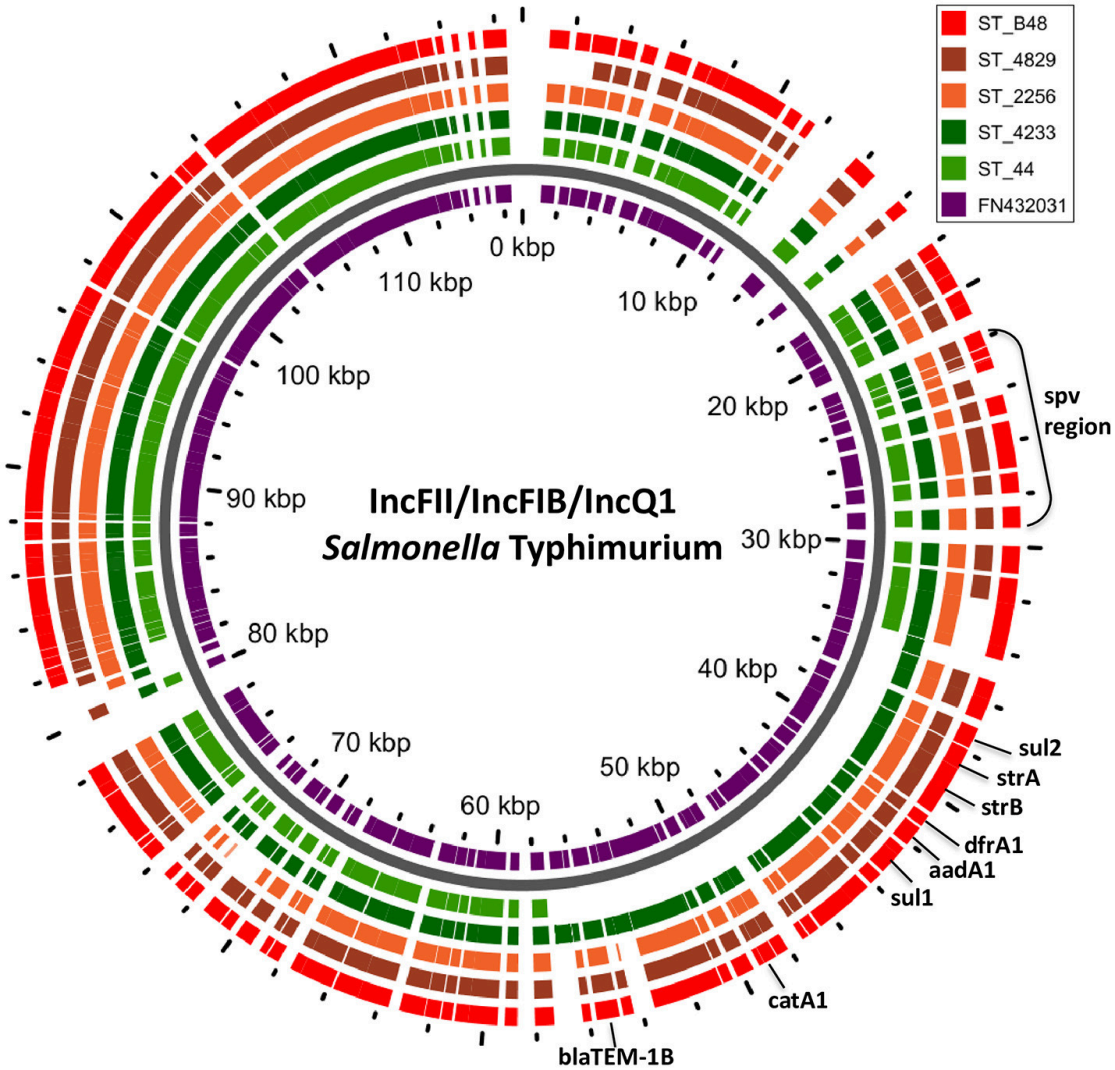
BLAST ring of IncFII(S)/IncFIB(S)/IncQ1 plasmid homologs of *Salmonella* Typhimurium and reference plasmid FN432031. Blast ring was created with GView. The middle ring is a reference plasmid, around which BLAST lanes are shown. Every lane corresponds to plasmid in each genome. The location of *spv* virulence-associated region, and antibiotic resistance genes are indicated outside the ring.

In *S*. Typhimurium, two plasmids were identified. One plasmid carried three plasmid replicon types (IncFII(S)/IncFIB(S)/IncQ1) and was found to be homologous (length coverage of 80–100%, sequence identity of 86–96%) to a previously characterized plasmid of *S*. Typhimurium of 117,047 bp in length (GenBank: FN432031). This plasmid carried *spv*-genes (Figure 2). The second one was of ColRNAI type. This plasmid was most similar (length coverage of 18–65%, sequence identity of 71–87%) to a plasmid of *S*. Typhi of 5,569 bp in length (GenBank: HQ730898). Two plasmids were detected in *S*. Virchow. The first plasmid carried three plasmid replicons (TrfA/IncHI2/IncHI2A) and was homologous (length coverage of 84%, sequence identity of 85%) to a plasmid from *S*. Typhimurium of 300,375 bp in length (GenBank: LN794248). The plasmid of *S*. Poona was also found to be most similar (length coverage of 100%, sequence identity of 87%) to the same plasmid. The second plasmid of *S*. Virchow carried a plasmid replicon of the IncX1 type and was homologous (length coverage of 100%, sequence identity of 94%) to a plasmid from *E. coli* of 38,611 bp in length (GenBank: JQ269336).

### Prediction of the antimicrobial resistance gene location in the plasmids

**Figure 3:**
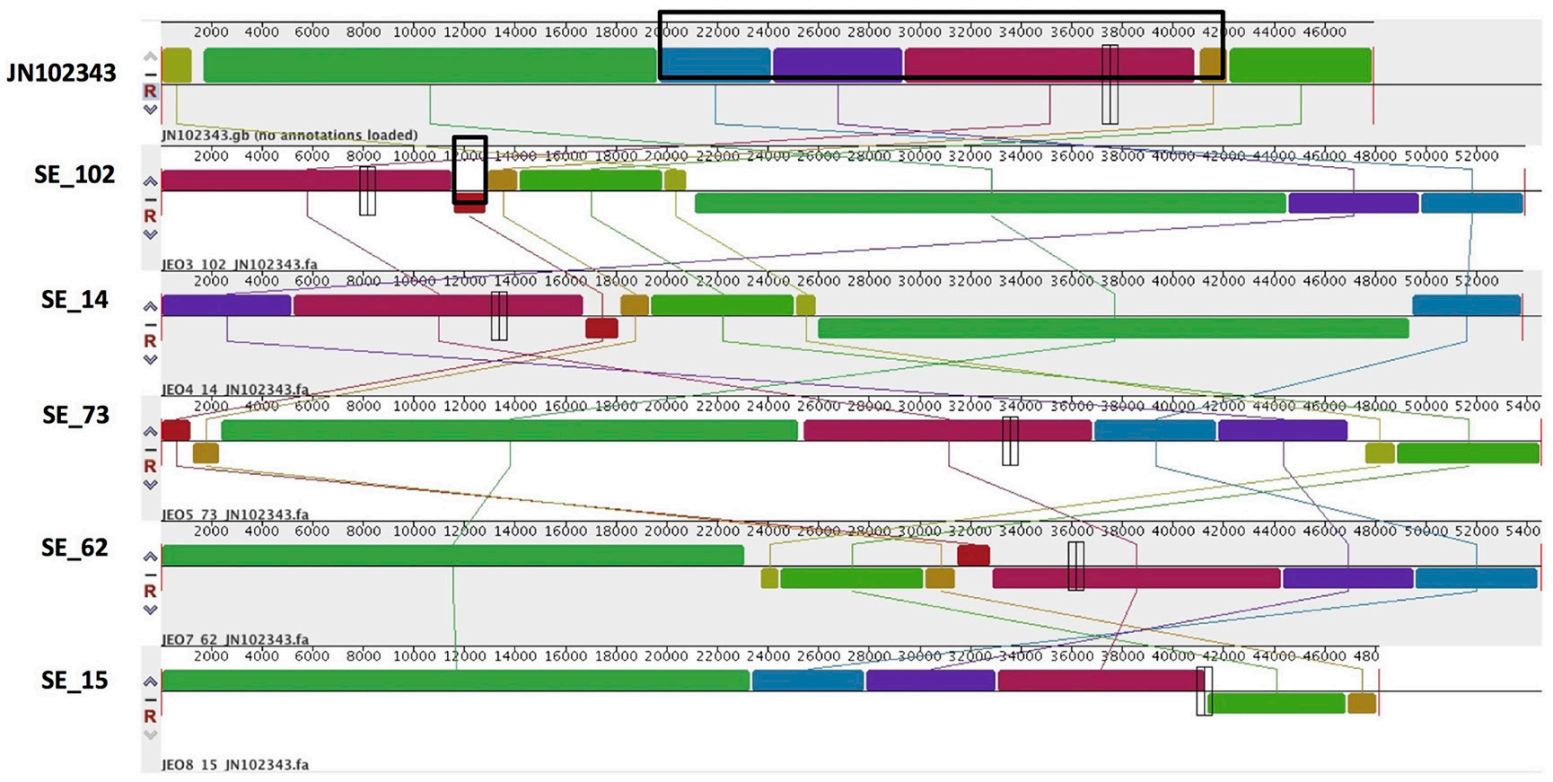
Alignment of IncN plasmid homologs of *Salmonella* Enteritidis to a reference plasmid JN102343. The alignment was created with MAUVE. The top row shows the reference plasmid, and the rows below the reconstructed plasmids in *Salmonella* Enteritidis from the study. Each of the colored blocks outlines a region of the genome sequence that aligned to part of another genome, and is presumably homologous. Areas with no blocks were not aligned and contain sequence elements specific to a particular genome. The black box in the reference plasmid surrounds a block found to contain antibiotic resistance genes *strA, strB, tet(A), blaTEM-1B* found in all isolates. The box in SE_102 indicate an insertion of *sul1, dfrA15*, which was found to be absent reference plasmid and in SE_15 isolate.

Combined analysis usingPlasmidFinder, ResFinder, plasmidSPAdes, and BlastN further allowed us to predict antimicrobial resistance gene location on the specific types of plasmids in all the 16 isolates under study (Tables 2, 3). In *S*. Enteritidis, the antimicrobial resistance genes were located on IncN plasmid (Figure 3). The transposable elements carrying resistance genes were inserted between the genes *nuc* and *fipA*. No transposon was found to be associated with the streptomycin resistance genes *strA* and *strB*, yet they are known to be the remnants of transposon Tn*5393* (Eikmeyer et al., 2012). As previously described, transposon Tn*1721* was linked to the carriage of tetracycline resistance genes *tetR* and *tetA*, and Tn*3* to the ampicillin resistance gene *blaTEM-1* (Eikmeyer et al., 2012). In addition, all isolates under study carried *sul1* and *dfr15*, which were not present in reference plasmid JN102343. Based on BlastN at NCBI, these genes and their flanking regions were most similar (length coverage of 100%, sequence identity of 100%) to the region found in *Enterobacter cloacae* plasmid (GenBank: CP021897) and with a lower similarity to the regions found in plasmids of other Enterobacteriaceae. The insertion of the region was associated with the XerC superfamily integrase. In the IncN replicon type negative *S*. Enteritidis isolate 113, the gene *bla*_TEM−1B_ gene was found to be located on an IncFII(S)/IncFIB(S) plasmid (Figure 1) associated to transposon Tn*3*.

In *S*. Typhimurium, resistance genes were carried on a IncFII(S)/IncFIB(S)/IncQ1 plasmid (Figure 2). Interestingly, *S*. Typhimurium strain 44 did not show resistance to multiple classes of antimicrobials, except for amino-penicillin and sulphometaxazole, and carried only plasmid replicons of the IncFII(S)/IncFIB(S) type, but not IncQ1. The BlastN analysis of this plasmid against the reference plasmid showed the absence of a whole contig carrying the IncQ1 replicon type, where resistance genes to various antimicrobials were present in all other isolates (Figure 2). In accordance with previous observations, all resistance genes found in the plasmid of *S*. Typhimurium ST313 isolates under study, were located within a composite Tn21-like transposon (Kingsley et al., 2009).

**Figure 4:**
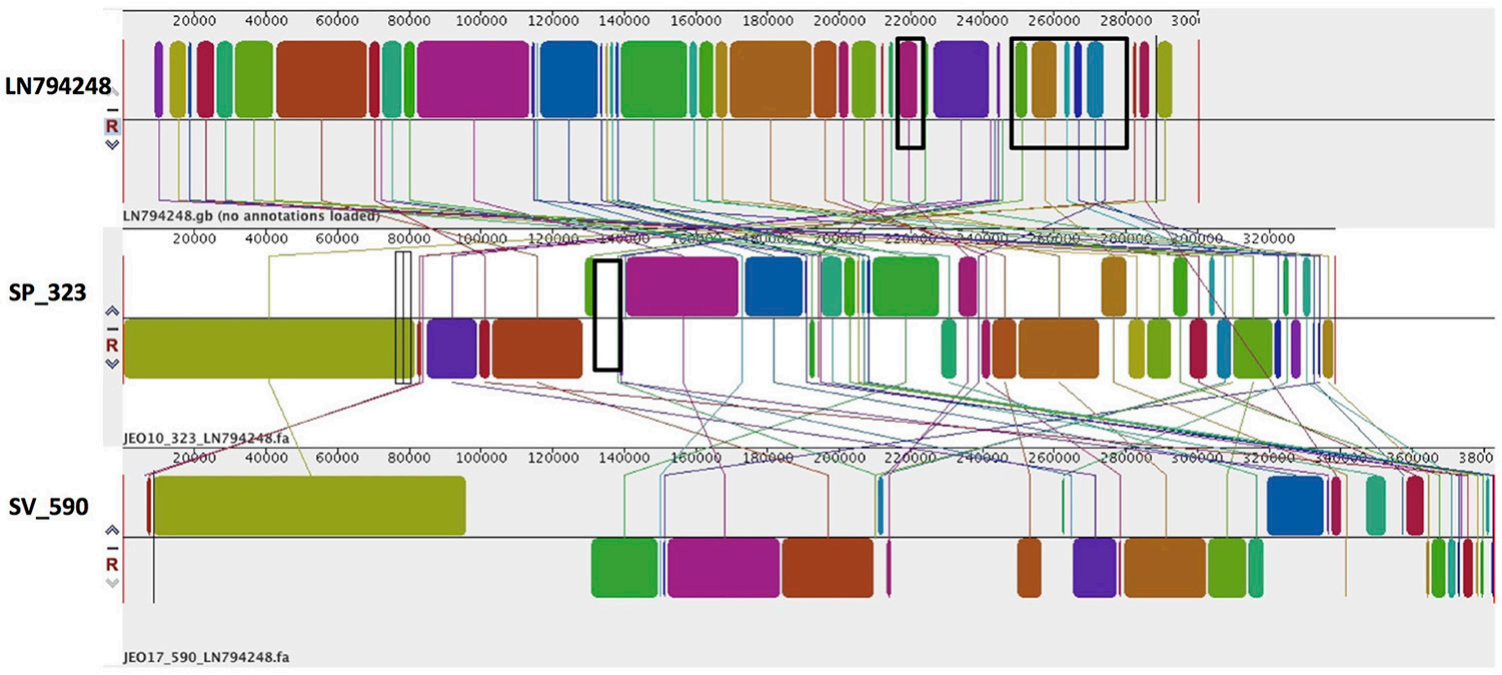
Alignment of TrfA/IncHI2/IncHI2A plasmid homologs of *Salmonella* Virchow and *Salmonella* Poona to a reference plasmid LN794248. The alignment was created with MAUVE. The top row shows the reference plasmid, and the rows below the reconstructed plasmids in *Salmonella* Virchow and *Salmonella* Poona from the study. Each of the colored blocks outlines a region of the genome sequence that aligned to part of another genome, and is presumably homologous. Areas with no blocks were not aligned and contain sequence elements specific to a particular genome. Black boxes in the reference plasmid surround blocks found to contain antibiotic resistance genes *strA, strB, blaTEM-1B, sul2, tet(A), blaCTX-M-15, aac(3)-Iia, catB3, aac(6')Ib-cr, blaOXA-1*, and *dfrA14*, which were present in *S*. Poona isolate from the study. The black box in the reconstructed plasmid in *S*. Poona indicates an insertion of *QnrB1*, which was absent in the reference plasmid.

As mentioned previously, a TrfA/IncHI2/IncHI2A plasmid was present in both *S*. Virchow and *S*. Poona strains (Figure 4), however only the plasmid found in *S*. Poona carried a multiple number of antimicrobial resistance genes including *bla*_CTX−M−15_. As previously mentioned this plasmid was homologous to a previously described IncHI2 plasmid of *S*. Typhimurium ST313 (GenBank: LN794248) isolated during an outbreak in Kenya (Kariuki et al., 2015). The majority of the resistance genes in this plasmid were located within a class 1 integron, while *bla*_CTX−M−15_ was associated with IS*Ecp1*. In addition to the resistance genes that were present in the reference plasmid, the plasmid of *S*. Poona under study carried the quinolone resistance gene, *qnrB1*. The *qnrB*1-encoding region was found to be most similar (length coverage of 100%, sequence identity of 99%) to a region within the plasmid of *Klebsiella pneumoniae* (GenBank: CP023922), and was associated with the transposon Tn*3*.

**Figure 5:**
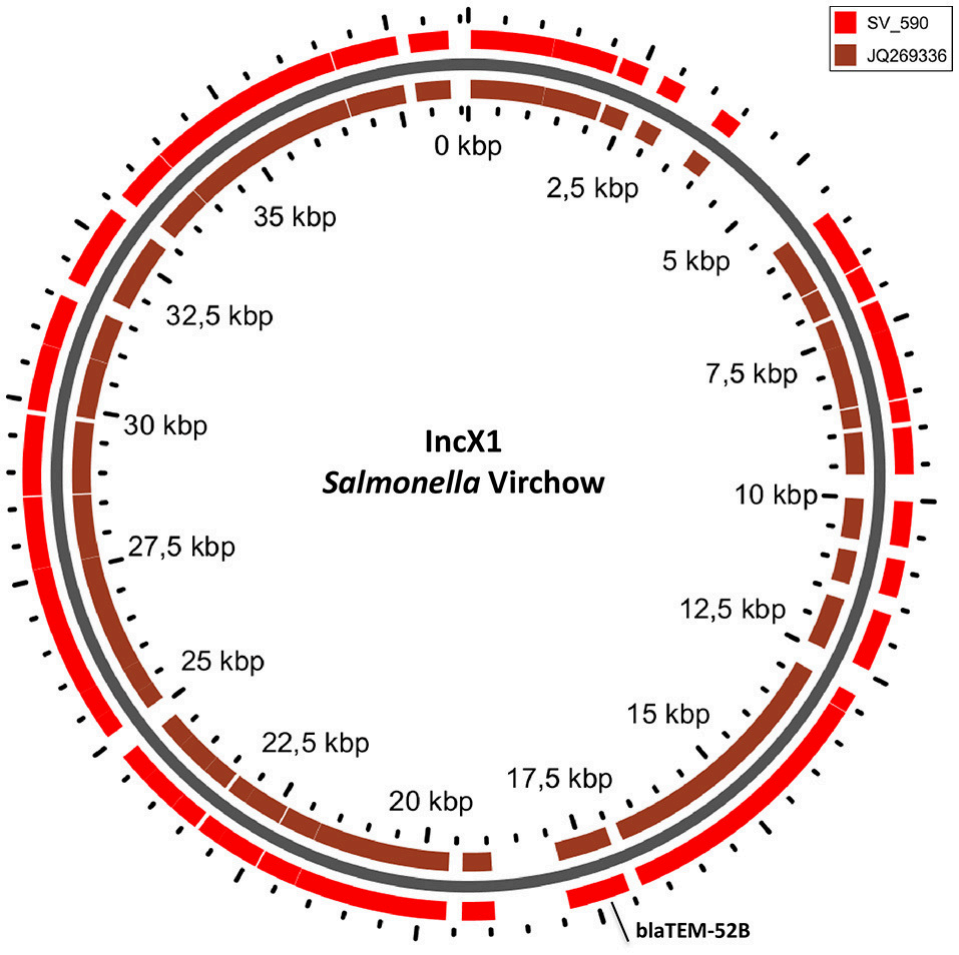
BLAST ring of IncX1 plasmid homolog of *Salmonella* Virchow and a reference plasmid JQ269336. Blast ring was created with GView. The middle ring is the reference plasmid, around which a BLAST lane of the plasmid in *S*. Virchow is shown. The location of antibiotic resistance gene is indicated outside the ring.

The only antimicrobial resistance gene identified in *S*. Virchow, *bla*_TEM−52B_, was located on the IncX1 plasmid linked to the transposon Tn*3* (Johnson et al., 2012) (Figure 5).

### S1-PFGE plasmid analysis

**Figure 6:**
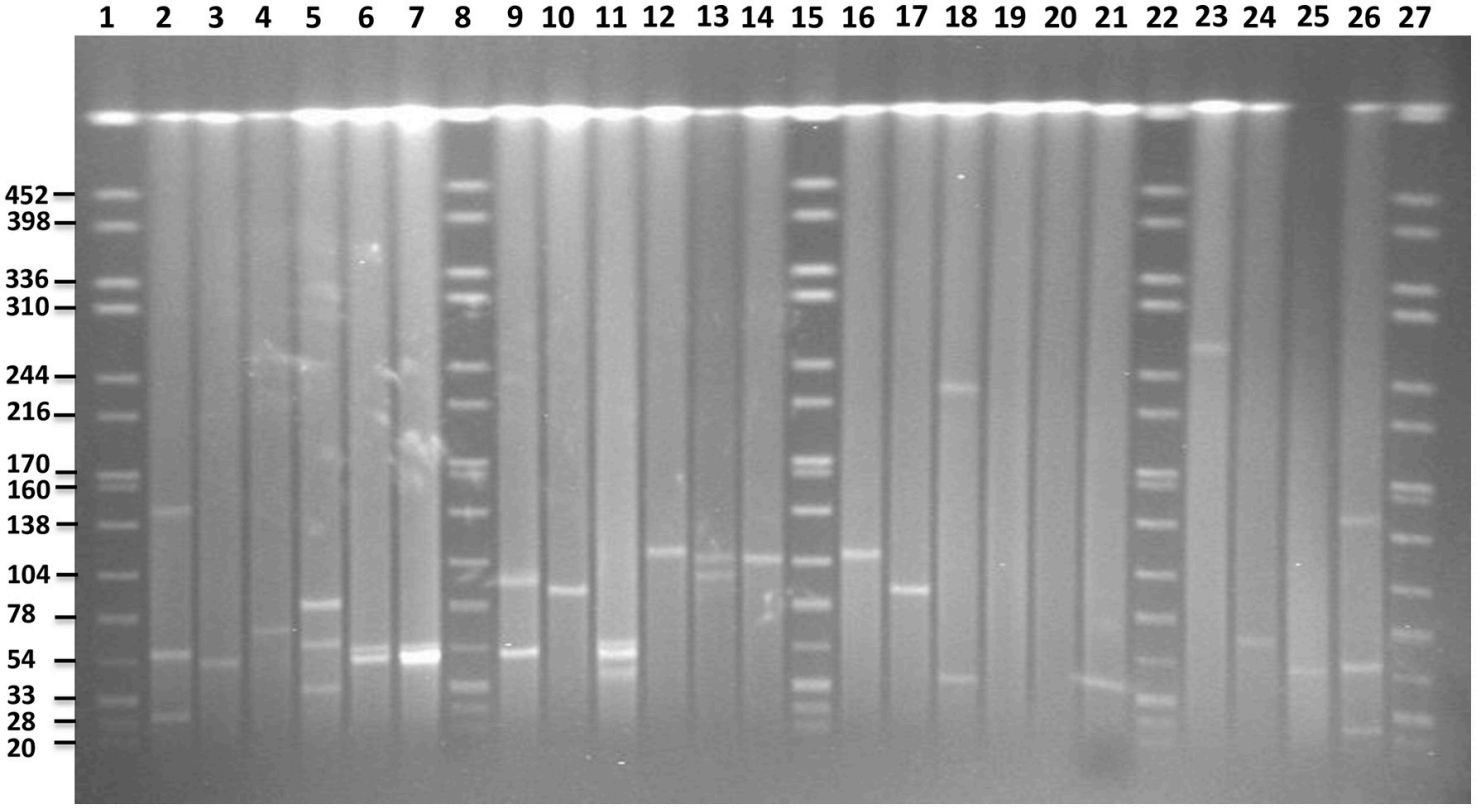
PFGE gel of plasmid profiles in the examined *Salmonella* serovars. Lanes 1, 8, 15, 22, and 27 represent fragments generated by *XbaI* digestion of *Salmonella* Braenderup strain H9812. Fragment sizes are indicated on the left side of the gel. Lanes 2, 3, 4, 24, 25, and 26 represent plasmids of known sizes in *Salmonella* isolates from our own collection. Lanes 5, 6, 7, 9, 10, and 11 represent plasmids profiles of *S*. Enteritidis isolates 113, 15, 102, 14, 73, and 62, respectively. Lanes 12, 13, 14, 16, and 17 represent plasmid profiles of *S*. Typhimurium isolates 4233, 2256, 4829, B48, and 44, respectively. Lanes 18, 19 represent plasmid profiles of *S*. Virchow 590 and 645, respectively. Lanes 20, 21 and 23 represent plasmid profiles of *S*. Colindale 4770, *S*. Oakland 114, and *S*. Poona 323, respectively.

S1-PFGE allowed visualization of plasmids with sizes ranging from 20 to 452 kb (Figure 6). The analysis revealed the presence of zero to up to three plasmids in the 16 strains characterized. In *S*. Enteritidis, the fragment of approximately 50 kb, corresponding to the IncN plasmid carrying resistance genes, was detected in 4 out of 5 isolates, which were shown to harbor this plasmid *in silico* (Table 2). According to the *in silico* prediction results, all six *S*. Enteritidis isolates carried the virulence plasmid IncFII(S)/IncFIB(S). In three of these strains, a plasmid of approximately 60 kb, corresponding to the size of the latter plasmid was present on the gel. A slightly bigger fragment of approximately 65–70 kb was found in the strain 113. According to our *in silico* results, this IncFII(S)/IncFIB(S) plasmid harbored the gene *bla*_TEM−1B_, which was absent in the IncFII(S)/IncFIB(S) plasmid from the three other strains. This resulted in an increase of the original virulence plasmid size, and it was classified as a virulence-resistance plasmid (Table 3). In the last two strains (73 and 14) of *S*. Enteritidis carrying the IncFII(S)/IncFIB(S) plasmid, the fragment of approximately 60 kb was not present, however a much larger fragment of approximately 100 kb was observed in both strains. Interestingly, two *S*. Enteritidis isolates carried additional plasmid fragments, which were not detected with the analysis *in silico*. Two such fragments of approximately 79 and 35 kb were found in strain 113, and one of approximately 50 kb in strain 62.

In four out of five isolates of *S*. Typhimurium, a fragment of approximately 120 kb corresponding to the *in silico* detected IncFII(S)/IncFIB(S)/IncQ plasmid carrying the *spv*-region and multidrug resistance genes was detected on the gel. In the isolate 44, shown to be lacking the IncQ part *in silico*, a smaller fragment of approximately 85 kb was present. In addition, one fragment of approximately 100 kb was found to be present in the strain 2256 using S1-PFGE, yet it was not found *in silico*.

In comparison to *in silico* analyses of the genomes, fragments corresponding to TrfA/IncHI2/IncHI2were found to be smaller using S1-PFGE. The presence of a fragment of approximately 300 kb in *S*. Poona (*n* = 1) and 230 kb in *S*. Virchow (*n* = 1) strains were detected. Besides, a fragment of approximately 40 kb corresponding to the IncX plasmid carrying the gene *bla*_*TEM*_**-_52*B*_ was observed in the *S*. Virchow isolate 590. Overall the findings were in agreement with the *in silico* analysis of these strains, and no more additional fragments were found with S1-PFGE. Plasmid fragments were also absent in the *S*. Oakland and *S*. Colindale strains.

## Discussion

### *Salmonella enterica* strains analyzed from ghana belong to common multidrug—associated MLST types

The majority of the *S*. Typhimurium strains in this study were assigned to ST313 (*n* = 4) with only one strain typed as ST19. *S*. Typhimurium ST313 has been reported to be the major cause of invasive disease in sub-Saharan Africa (Kingsley et al., 2009). Recently, it has been also detected in India and Scotland (Crump et al., 2015). The reason behind an increased pathogenicity of ST313 strains compared to other isolates of *S*. Typhimurium is unclear, yet it may be associated with structural changes in their genome resulting in better host specialization or invasiveness (Crump et al., 2015). In addition, many *S*. Typhimurium non-typhoidal invasive isolates, including ST313, exhibit a multidrug resistance phenotype, which is encoded in the virulence plasmid of this serovar. It was suggested that antimicrobial usage may be selecting for the maintenance of these virulence-resistance plasmids in such isolates (Herrero et al., 2008; Kingsley et al., 2009). The *S*. Typhimurium strain assigned to ST19 did not exhibit a multidrug resistance pattern, and did not carry antimicrobial resistance genes. The ST19 genotype is also a common sequence type observed in Africa, however it is related to classical gastroenteritis, with no reported invasive infections (Kingsley et al., 2009; Boyle et al., 2011; Okoro et al., 2012). The fact that our study has an over-representation of ST313 strains may be related to the selection of strains in the original study (Andoh et al., 2017), which included a disproportional number of isolates from hospitalized patients, and thus were likely to originate from patients with systemic salmonellosis.

All isolates of *S*. Enteritidis were assigned to ST11, which is the most common genotype associated to this serovar (Achtman et al., 2012), and which is present in both humans and food production animals globally (Ghaderi et al., 2015; Papadopoulos et al., 2016). Invasive variants of *S*. Enteritidis are increasingly reported in developing countries and have been associated with virulence plasmids carrying multidrug resistant genes (Rodríguez et al., 2012). The association of IncI or IncN plasmids with the presence of cephalosporin and fluoroquinolones resistance determinants have been also shown recently in several clones worldwide (Antunes et al., 2011; Bado et al., 2012; Wong et al., 2015).

A strain of *S*. Virchow was assigned to the sequence type ST16. This type was not listed in the original publication on MLST by Achtman et al. (2012), but was later described as the most common ST linked to this serovar in a large collection of ESBL producing *S*. Virchow strains from Korea (Kim et al., 2016). A strain of *S*. Colindale was assigned to ST584. According to the original publication on *Salmonella* MLST (Achtman et al., 2012), this ST is one out of the two associated to this serovar. Finally, the strain of *S*. Poona was assigned to the genotype ST308 which is one out of six STs related to this serovar in the original MLST database (Achtman et al., 2012). So far, there have been no reports on the association of the latter serovar with plasmid mediated multidrug or cephalosporin resistance.

### Multiple resistance genes present in *Salmonella* isolates

The majority of the strains analyzed were resistant to more than one antimicrobial. Multidrug resistant *Salmonella* has been increasing worldwide over the years, and has been mostly associated with the acquisition of β-lactamases encoded on mobile genetic elements. These plasmid-mediated resistances have been detected in Europe, USA and some countries in Asia and Africa (Valenzuela de Silva et al., 2006; Sjölund-Karlsson et al., 2011; Barua et al., 2013; Obeng-Nkrumah et al., 2013).

The majority (*n* = 12/16) of the amino-penicillin and suspected ESBL positive strains in our study, harbored *bla*_TEM_ genes only. The *bla*_TEM−1B_ and *bla*_TEM−52B_ genes were found in 11 and one *bla*_TEM_ positive strains, respectively. *bla*_TEM−1B_ hydrolyses narrow spectrum cephalosporins, cefamandole, cefoperazone and penicillin (Fonzé et al., 1995), and is the most common beta lactamase encoding-gene among Enterobacteriaceae (Briñas et al., 2002; Olesen et al., 2004).

The gene *bla*_CXT−M−15_ was detected in only one cephalosporin resistant *S*. Poona strain. *Salmonella* isolates harboring *bla*_CTX−M_ genes have not been previously reported in humans or food animals in Ghana, but they have been reported in other regions of the world (Barua et al., 2013; Akinyemi et al., 2014). Strains positive for *bla*_CTX−M−15_ have been associated with bloodstream infections in children and patients from intensive care units in Tanzania (Blomberg et al., 2005; Ndugulile et al., 2005) Senegal (Weill et al., 2004) and in other countries (Karim et al., 2001; Mushtaq et al., 2003; Pagani et al., 2003; Tawfik et al., 2011).

Plasmids carrying *bla*_CTX−M_ genes often harbor genes encoding resistance to other antimicrobials (aminoglycosides, tetracycline, sulphonamides, trimethoprim and quinolones) (Diop et al., 2016) and therefore may confer co-resistance. This is also the case for the *S*. Poona strain under study, which was shown to carry *bla*_CTX−M−15_ together with genes encoding resistance to trimethoprim, sulphonamide, ampicillin and tetracycline on the same plasmid.

Interestingly, the plasmid-associated antimicrobial resistance gene profile varied between strains harboring the same plasmid type. In all cases, the plasmids regions carrying antimicrobial resistance genes were located within other mobile genetic elements such as transposons and integrons. This suggests that different recombination processes linked to mobile genetic elements might have occurred leading to acquisition and/or loss of antimicrobial resistance determinants in the plasmids as previously shown (Herrero-Fresno et al., 2014; García et al., 2016).

### Distinct plasmids carry antimicrobial resistant genes in different *Salmonella* serovars

Virulence-associated FII(S)/IncFIB(S)/IncQ plasmids carrying multidrug resistance were identified in four isolates of *S*. Typhimurium belonging to ST313. This kind of virulence-resistance plasmids were identified in previous studies (Guerra et al., 2002; Villa et al., 2010). The ST313-associated plasmid, pSLT-BT (EMBL: FN432031) (Kingsley et al., 2009), was also found in the ST313 isolates analyzed in this study where the MDR genes were located on a composite Tn*21*-like transposon, as was the case in previous investigation of ST313 strains (Kingsley et al., 2009). Interestingly, one *S*. Typhimurium isolate assigned to ST19 harbored a pSLT-BT-like plasmid as well, however the strain was sensitive to all antibiotics tested, and the region containing the IncQ replicon together with all the resistance genes was missing. This suggests that this region may be specific to ST313, and it would be of relevance to investigate whether this specific pSLT-BT region contributes to its invasive phenotype. Only one ST19 isolate was analyzed in our study, and the lack of such region should be confirmed in plasmids from a larger collection of non-invasive *S*. Typhimurium isolates.

In the *S*. Enteritidis strains under study, multiple resistance genes were located on a IncN plasmid. All the six *S*. Enteritidis isolates analyzed were assigned to ST11, but one of them did not carry the multidrug resistant IncN plasmid. Instead, only the *bla*_*TEM*_ gene was located on the FII(S)/IncFIB(S) *S*. Enterititids virulence plasmid (Allard et al., 2013). *S*. Enteritidis ST11 is highly prevalent worldwide and is associated with genetically diverse isolates. Based on this study and on previous reports, it appears that multidrug resistance in ST11 strains can be located on both virulence-associated plasmids and on non-virulence plasmids of other replicon types, and this may be geographical location or ST11 sub-lineage dependent (Antunes et al., 2011; Bado et al., 2012; Rodríguez et al., 2012; Wong et al., 2015).

In our study, the *S*. Poona isolate carried multiple resistance genes (including *bla*_CTX−M−15_) on a TrfA/IncHI2/IncHI2A plasmid that has not been previously described in this serovar. IncHI2 plasmids carrying the *bla*_CTX−M−15_ gene have been reported in *S*. Virchow strains from South Korea (Kim et al., 2016). IncHI2 plasmids have also previously been associated with carriage of *bla*_CTX−M_ genes in *S*. Typhimurium ST313 strains in Kenya (Kariuki et al., 2015), and in *S*. Virchow in the UK (Hopkins et al., 2006). A TrfA/IncHI2/IncHI2A plasmid similar to the one observed in *S*. Poona, however with no antimicrobial resistance genes, was also detected in the other phenotypically cephalosporin and ampicillin resistant *S*. Virchow isolate. In this *S*. Virchow isolate, the only antimicrobial resistance gene, *bla*_TEM−52B_, was found on an IncX1 plasmid. The plasmid of IncX type was previously reported to be highly associated with the carriage of *bla*_TEM−52B_ gene sometimes together with resistance genes to other antimicrobials in a diverse *Salmonella* serovars from animals, meat products and humans in Europe, Canada and Korea (Bielak et al., 2011).

A combination of bioinformatics tools and S1-PFGE used in the present study enabled nearly full mapping of antibiotic resistance plasmids in different *Salmonella enterica* serovars. In most cases *in silico* prediction was concordant with the findings by S1-PFGE, however, there were some exceptions in particular related to possible plasmid fusion events. Thus, in two cases, *in silico* prediction and S1-PFGE analysis showed that either the IncFII(S)/IncFIB(S) or the IncN plasmid may have fused with other plasmids in two *S*. Enteritidis strains (73 and 14). In strain 73, the IncN and the IncFII/IncFIB were found to be fused *in silico*, and in agreement with this, only one fragment corresponding to the size of both plasmids was found in the S1-PFGE analysis. It is generally expected that after the fusion the loss of transfer genes occurs, however, blastN did not show differences in conjugal regions of these plasmids in comparison to these regions in the reference plasmids. In strain 14, a fragment of approximately 55 kb was present together with the fragment of 100 kb. According to *in silico* results, no indication of IncFII/IncFIB and IncN fusion was detected. Due to this, it remains unclear what kind of insertion/fusion occurred in the plasmids within this strain. However, the loss of two conjugative genes (TraM and X) were detected in the IncF plasmid of this strain in comparison to reference plasmid, which indicated the possible fusion event (García et al., 2007). Presently there is no method which could confirm these results from WGS (Arredondo-Alonso et al., 2017; Orlek et al., 2017), therefore only the sequencing of purified plasmids would enable to confirm plasmid fusion in these strains.

Finally, it is important to note that the approach that has been used in this study has a number limitations. Firstly, the library construction for sequencing of the genomes in the study was performed using Nextera XT kit, which includes a PCR-enrichment step, which biases the coverage of the reads belonging to different parts of the genome, and thus interferes with the ability of plasmidSPAdes to detect plasmids in some isolates. To overcome this limitation, blastN against reference and MAUVE were used for plasmid detection in such isolates. However, the method could be improved by using PCR-free sequencing protocols.

In summary, in the present study we have characterized plasmids from multidrug resistant *Salmonella* strains from Ghana. In doing so, we have demonstrated the successful implementation of a systematic approach to characterize plasmids directly from whole genome sequences and to predict whether antimicrobial resistance genes are located on such plasmids. In addition, previously unreported resistant or virulence-resistant plasmids in different *Salmonella* serovars were identified. This approach may be very valuable in future studies on how plasmids contribute to the development of novel R-plasmids and on plasmid evolution. The results of the detailed mapping of the few strains elucidate that homologous plasmids may be found in different bacteria originating from different regions of the world, and also that specific plasmid types may be associated with the antibiotic resistance in different *Salmonella* strains and serovars.

## Author contributions

JO, AD, and KO-D contributed conception and design of the study; EK performed bioinformatic analysis and wrote the first draft of the manuscript; LA performed ESBL testing and wrote the sections of the manuscript; SA prepared samples for whole genome sequencing; AH-F wrote the sections of the manuscript. All authors contributed to manuscript revision, read and approved the submitted version.

### Conflict of interest statement

The authors declare that the research was conducted in the absence of any commercial or financial relationships that could be construed as a potential conflict of interest.

## Footnotes

^1^http://www.cbs.dtu.dk/services/.

^2^http://www.pulsenetinternational.org/protocols/.
