# Supplementary material for: The Use of a Combined Bioinformatics Approach to Locate Antibiotic Resistance Genes on Plasmids From Whole Genome Sequences of Salmonella enterica Serovars From Humans in Ghana
Source: Front Microbiol. 2018 May 17;9:1010. doi: 10.3389/fmicb.2018.01010 (PMC5966558; doi:10.3389/fmicb.2018.01010)
Supplement: Supplementary file 1 [file Data_Sheet_1.pdf]

# *Supplementary Material*

# Improved identification of multidrug resistance plasmids in whole genome sequences of *Salmonella enterica* serovars from humans in Ghana

**E. Kudirkiene, L. A. Andoh, S. Ahmed, A. Herrero-Fresno, A. Dalsgaard, K. Obiri-Danso, J. E. Olsen***

***Correspondence:** John Elmerdahl Olsen: jeo@sund.ku.dk

## 1. Graphic representation of systematic approach for plasmid reconstruction analysis in one selected *Salmonella* isolate under study.

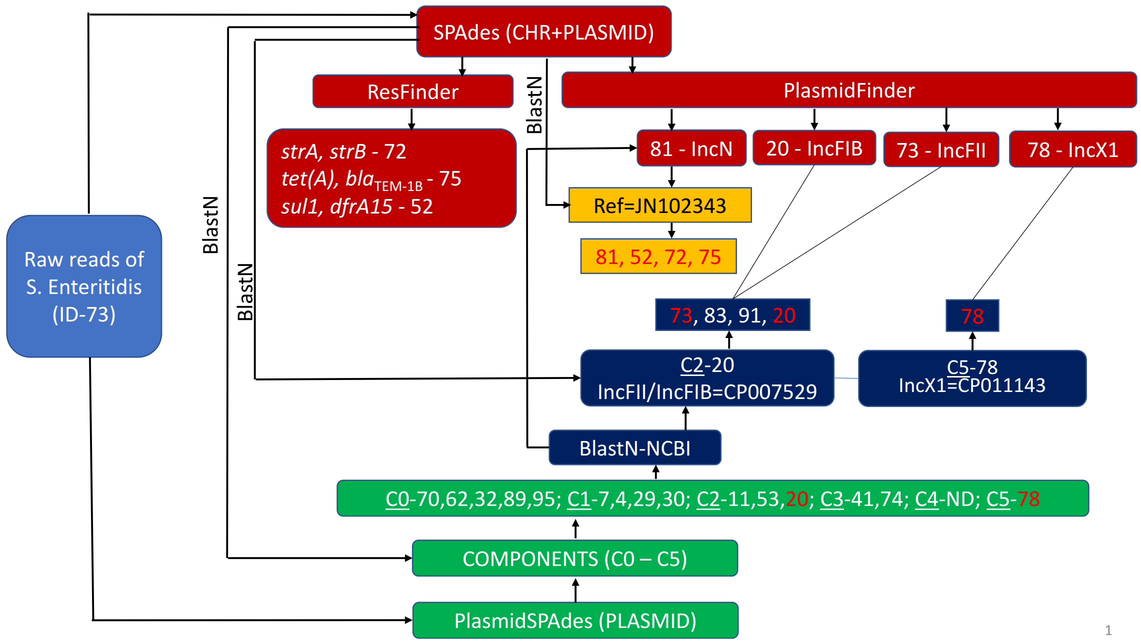


**Figure 1.** The light blue box indicate the beginning of the analysis. The raw reads were simultaneously assembled with two methods available in SPAdes assembly program. In this example, the green boxes show that in total five putative plasmids (indicated as C0-C5) were identified in the isolate using plasmidSPAdes assembly. The red boxes show the output of ResFinder and PlasmidFinder tools which were applied on the genomes assembled with SPAdes. The location of resistance genes and plasmid replicons on specific contigs is also indicated. The dark blue boxes show the results of BlastN analysis either on NCBI or locally, and the resulting output such as reference plasmids, and their location in the whole genome assembly generated by SPAdes. Contig numbers coloured in red are the ones to be found associated with the plasmids and the resistance genes.

**2. Serovar and sequence type of *Salmonella* isolates under study as predicted *in silico* using SeqSero and SISTR online analysis tools**

**Table 1**.

| Strain ID | Phenotypic assignment | SeqSero | | SISTR | | | | | | |
|---|---|---|---|---|---|---|---|---|---|---|
| | | Antigenic profile (O:H1:H2) | Serotype | Serovar (overall) | Serovar (cgMLST) | Serogroup | H1 | H2 | Serovar (MLST) | MLST ST |
| 4770 | *S.* Colindale | 13:r:1,7 | N/A | Colindale | Virchow | C1 | r | 1,7 | | 584 |
| 102 | *S.* Enteritidis | 9:g,m:- | Enteritidis | Enteritidis | Enteritidis | B | g,m | - | Enteritidis | 11 |
| 14 | *S.* Enteritidis | 9:g,m:- | Enteritidis | Enteritidis | Enteritidis | D1 | g,m | - | Enteritidis | 11 |
| 73 | *S.* Enteritidis | 9:g,m:- | Enteritidis | Enteritidis | Enteritidis | D1 | g,m | - | Enteritidis | 11 |
| 113 | *S.* Enteritidis | 9:g,m:- | Enteritidis | Enteritidis | Enteritidis | B | g,m | - | Enteritidis | 11 |
| 62 | *S.* Enteritidis | 9:g,m:- | Enteritidis | Enteritidis | Enteritidis | D1 | g,m | - | Enteritidis | 11 |
| 15 | *S.* Enteritidis | 9:g,m:- | Enteritidis | Enteritidis | Enteritidis | B | g,m | - | Enteritidis | 11 |
| 114 | *S.* Oakland | 13:z:- | II 13,22:z:- | None:z:1,6 | Newport | | z | 1,6 | Oakland | 605 |
| 323 | *S.* Poona | ?:z:1,6 | N/A | None:z:1,6 | Poona | | z | 1,6 | | 308 |
| 2256 | *S.* Typhimurium | 4:i:1,2 | Typhimurium | Typhimurium | Typhimurium | B | i | 1,2 | Typhimurium | 313 |
| 4233 | *S.* Typhimurium | 4:i:1,2 | Typhimurium | Typhimurium | Typhimurium | B | i | 1,2 | Typhimurium | 313 |
| 4829 | *S.* Typhimurium | 4:i:1,2 | Typhimurium | Typhimurium | Typhimurium | B | i | 1,2 | Typhimurium | 313 |
| B48 | *S.* Typhimurium | 4:-:- | N/A | B:None:- | Typhimurium | B | | - | Typhimurium | 313 |
| 44 | *S.* Typhimurium | 4:i:1,2 | Typhimurium | None:i:1,2 | Typhimurium | | i | 1,2 | Typhimurium | 19 |
| 590 | *S.* Virchow | 30:-:- | N/A | None:r:1,2 | Virchow | | r | 1,2 | Virchow | 16 |
| 645 | *S.* Virchow | 30:r:1,2 | Grandhaven | None:r:1,2 | Virchow | | r | 1,2 | Virchow | 359 |

N/A – data is not available
